# Brazilian pediatricians’ adherence to food allergy guidelines—A cross-sectional study

**DOI:** 10.1371/journal.pone.0229356

**Published:** 2020-02-24

**Authors:** Sarah Cristina Fontes Vieira, Victor Santana Santos, Jackeline Motta Franco, Hiram Menezes Nascimento-Filho, Kamilla de Oliveira e Silva Solis Barbosa, Divaldo Pereira de Lyra-Junior, Kleyton de Andrade Bastos, Rosana Cipolotti, Mônica Lisboa Chang Wayhs, Mário César Vieira, Dirceu Solé, Mauro Batista de Morais, Ricardo Queiroz Gurgel

**Affiliations:** 1 Graduate Program in Health Science, Federal University of Sergipe, Aracaju, Sergipe, Brazil; 2 Reference Center for Food Allergy of Sergipe, Federal University of Sergipe, Aracaju, Sergipe, Brazil; 3 Department of Medicine, Federal University of Sergipe, Aracaju, Sergipe, Brazil; 4 Department of Nursing, Federal University of Alagoas, Arapiraca, Alagoas, Brazil; 5 Department of Pediatrics, Federal University of Santa Catarina, Florianópolis-Santa Catarina, Brazil; 6 Center for Pediatric Gastroenterology, Hospital Pequeno Príncipe, Curitiba, Paraná, Brazil; 7 Department of Pediatrics, Pontifical Catholic University of Paraná, Curitiba, Paraná, Brazil; 8 Department of Pediatrics, Federal University of São Paulo, São Paulo, São Paulo, Brazil; Universidad de Antioquia, COLOMBIA

## Abstract

Food allergy is an emerging clinical condition in pediatrics, so recommendations on its management have been widely published. Studying pediatricians’ adherence to these clinical practice guidelines (CPG) and understanding the reasons for their non-compliance can help to promote better management of this condition. A cross-sectional study was conducted by a survey among Brazilian pediatricians, randomly selected during the 38^th^ Brazilian Congress of Pediatrics, which took place in October, 2017. A validated questionnaire with 16 questions addressing knowledge and practice on food allergy, as well as self-reported adherence to international guidelines was applied. Of the total of 415 pediatricians from all regions of the country who were surveyed, only 69 (16.7%) had a satisfactory adherence rate (≥80%). Adequate adherence to the guidelines was associated with the variables: ‘evaluating more than 10 children with suspected cow’s milk allergy (CMA) per month'; 'having read the Brazilian consensus'; or 'being aware of any international food allergy guideline'. In 8 of the 10 questions that assessed conscious adherence, a minority of those surveyed (20.3–42.3% variation) stated that they knew that their response was in line with the guidelines. This finding was statistically significant (p<0.05) in 7 of these 8 questions. The self-reported adherence of Brazilian pediatricians to international food allergy guidelines was low. Pediatricians who evaluated a higher number of children with suspected CMA or who were aware of the recommendations, had a higher rate of adherence. The results of the survey found that lack of resource was the major reported barrier to guideline adherence but lack of awareness must be a relevant non perceived barrier. This study shows the pediatricians´ self-reported adherence to food allergy guidelines in a widely overview for the first time in Brazil. More studies are necessary to investigate adherence to guidelines by pediatricians in other countries and to develop strategies to improve adherence.

## Introduction

Food allergy is common in the pediatric population and may cause nutritional, emotional and socioeconomic impact to patients, their families, and society [1,2]. The global prevalence of food allergies has been increasing and has reached up to 10% of the population [3,4]. Although epidemiological data are scarce in Brazil, a study performed in the 5 different geographical regions of the country by 30 pediatric gastroenterologists estimated the incidence of suspected cow’s milk allergy (CMA) to be 2.2% and its prevalence 5.4% [5]. Gonçalves (2016) and colleagues [6] found that 23.5% of parents reported food allergies in infants but only 1.9% were confirmed after clinical evaluation and tests, including oral food challenge (OFC) when necessary, with cow’s milk being the major food allergy. Correctly diagnosing and managing food allergy is still a challenge.

Clinical practice guidelines (CPG) have been published to improve the quality of care and to standardize the treatment of children with suspected or diagnosed food allergy [7–16]. However, adherence to these guidelines in health care is often low [17]. Therefore, it is imperative to elaborate strategies for implementing guideline recommendations in clinical practice [18–20].

In Brazil, different studies have demonstrated that knowledge about food allergy management amongst health care professionals may be inadequate [21–24]. However, the adherence of Brazilian pediatricians to the guidelines as well as the reasons for possible non-compliance with the recommendations are still unknown. Furthermore, the available recommendations for food allergy in Brazil do not fulfill the methodological criteria required to be classified as guidelines [25]. This study, therefore, aimed to evaluate the adherence of Brazilian pediatricians to food allergy CPG and the possible reasons for not putting them into practice.

## Methods

### Study design

A survey was conducted among Brazilian pediatricians during the 38th Brazilian Congress of Pediatrics in 2017, to evaluate their knowledge about the prevention, diagnosis, and treatment of food allergy, in addition to their self-reported adherence to international guidelines [7–10,12–16]. Pediatricians in the area of the congress hall were randomly invited to participate in the study and after agreeing to take part in the study completed a written Informed Consent Form, they anonymously completed a paper-based questionnaire. The study was approved by the Research Ethics Committee of the Federal University of Sergipe, under registration number 70282117.2.0000.5546 and the Brazilian Society of Pediatrics, the organizer of the event. The ethical principles established by the Declaration of Helsinki were followed.

### Population and sample

The sample size was calculated based on the number of Brazilian pediatricians registered at the Brazilian Society of Pediatrics (n = 23,042). We assumed that 50% of pediatricians would adhere to ≥80% of CPG recommendations, with a confidence interval of 95% and maximum error of 5%, which resulted in a minimum sample size of 378 pediatricians. However, to minimize any bias for dropouts, we added 10% to the sample size. We therefore enrolled 415 participants.

All pediatricians attending the congress were eligible and were randomly invited to participate in the research, completing the questionnaire in the congress hall area. Only professionals who had received formal training in pediatrics and who were practicing the specialty in Brazil were included. Other health care professionals and pediatricians working outside of Brazil were not included in the study.

### Questionnaire

A questionnaire with 16 questions was developed (see supplement), with 14 multiple choices and 2 open-ended questions. Among the 16 questions, 13 of the multiple-choice questions evaluated the pediatrician’s knowledge and practices in relation to food allergy prevention, diagnosis and treatment and 3 assessed whether the pediatrician was familiar with the Brazilian consensus (yes or no) and any international guidelines (yes or no) for food allergy, as well as their reasons for non-compliance with current recommendations. The last two questions were open-ended, and the respondents were asked to state which international guidelines they were familiar with and what were the reasons for any intentional non-compliance with the recommendations. Among the 13 multiple choice questions that investigated knowledge and practice, 10 evaluated the management in situations for which there are well-established recommendations in international guidelines [7–10,12–16] and that are the same in the Brazilian consensus [25]. These 10 questions assessed self-reported adherence to the CPG in respect of the following: identification of risk factors for food allergy; differentiation between anaphylaxis and food protein induced enterocolitis syndrome (FPIES); diagnosis of cow’s milk allergy (CMA) with late onset gastrointestinal manifestations; recognition of food protein induced allergic proctocolitis (FPIAP); recommendation for oral food challenge (OFC) for diagnosis of CMA; timing to OFC (evaluation of tolerance development); how is complementary feeding introduced in infants with CMA; appropriate indications for soy formula; indication of extensively hydrolyzed protein formula as the first option to substitute or complement breastmilk in CMA; and when prescribing calcium supplement in CMA. In order to evaluate conscious adherence, after responding each of the multiple-choice questions, pediatricians were asked whether they thought their practice was in agreement or not with the guidelines, or if they did not know if their approach would follow the recommendations. For content validation of the survey, the Delphi technique was used with 6 experts in the field, in five rounds [26]. In a pilot study prior to the survey at the conference, its applicability was evaluated by 34 pediatricians from the 5 regions of the country. The sociodemographic data of the surveyed individuals and their professional profiles were collected together with the paper-based questionnaire to identify variables associated with adherence.

### Outcomes

The primary outcome of the study was the self-reported adherence of pediatricians to published CPG for food allergy. The adherence score was calculated based on the number of questions correctly answered out of the 10. The correct answer was the one that was in accordance with what is recommended in the international guidelines for food allergy. For these ten questions, international and Brazilian guidelines present similar recommendations and, therefore, the same answers to the questions.

As secondary endpoints, we evaluated knowledge and practice in respect of food allergy, having read the national consensus and awareness of any international guidelines, conscious adherence, and reasons for intentional non-compliance with the guidelines.

### Data analysis

Categorical variables were described using frequencies and percentages. Multiple comparisons were analyzed by using Z-test statistics. When a Z-test was significant, we performed multiple comparisons using the Bonferroni test (post-hoc test) to determine differences between the groups.

Factors associated with adherence were established using the odds ratio (OR) with 95% confidence intervals (CI). Considering that a cutoff point to indicate adequate adherence to guidelines is not established and must vary among different clinical practice scenarios, we assumed an 80% cutoff to define adherence and non-adherence to guideline. Logistic regression was used to identify factors independently associated with adherence. P values <0.20 were used to select variables for inclusion in the logistic regression and we identified covariates that had significant bivariate tests. Backwards stepwise modeling was used, removing covariates if their statistical significance was lost (P >0.05) or if the variable was not a confounder through its effect on other parameters in the models. Data were analyzed using SPSS version 23.0 (IBM Corporation, Armonk, NY).

## Results

A total of 415 (7.9%) pediatricians from the 5246 participants at the 38th Brazilian Congress of Pediatrics, representing 1.8% of the 23,042 members of the Brazilian Society of Pediatrics, were surveyed. The characteristics of the pediatricians included in the study are detailed in Table 1.

**Table 1:** Characteristics of surveyed pediatricians.

VARIABLES	N (%)
**Age, median (IQR)**	39 (32–52)
**Region**	
North	42 (10.1)
Northeast	145 (34.9)
West Central	29 (7.0)
Southeast	165 (39.8)
South	34 (8.2)
**Time since conclusion of pediatric residency training (years), median (IQR)**	10 (2–22)
**Time since conclusion of pediatric residency training (years)**	
0–5 years	155 (37.3)
6–10 years	63 (15.2)
>10 years	196 (47.2)
**Work Setting**	
**Private medical clinic**	
Yes	217 (52.3)
No	198 (47.7)
**Public health service**	
Yes	266 (64.1)
No	149 (35.9)
**Hospital**	
Yes	273 (65.8)
No	142 (34.2)
**Neonatal service**	
Yes	70 (16.9)
No	345 (83.1)
**Evaluation of children with suspected CMA**	
Yes	302 (72.8)
No	113 (27.2)
**Number of children with suspected CMA evaluated per month**	
0–5 children	207 (49.9)
6–10 children	51 (12.3)
>10 children	23 (5.5)

CMA, cow´s milk allergy; IQR, interquartile range

*For ‘Time since conclusion of pediatric residency training’ and ‘Number of children with suspected CMA evaluated per month’ there were incomplete data with a total of 414 and 281 responses, respectively.

None of the pediatricians fully adhered to international food allergy guidelines. Only 69 (16.7%) achieved an adherence rate of ≥80%, and there was no statistically significant difference among pediatricians practicing in different regions of the country (Table 2).

**Table 2:** International guideline adherence rate ≥80% by Brazilian region.

Region	Adherence rate ≥80%	
	Yes (%)	No (%)
North	4 (9.5)^a^	38 (90.5)^a^
Northeast	31 (21.7)^a^	112 (78.3)^a^
West Central	7 (24.1)^a^	22 (75.9)^a^
Southeast	23 (13.9)^a^	142 (86.1)^a^
South	4 (11.8)^a^	30 (88.2)^a^

Z-test with Bonferroni correction. Each superscript letter denotes a subset of **Adherence rate ≥80%** categories whose proportions do not differ significantly from each other at the 0.05 level.

### Guideline adherence awareness

A total of 140 (33.7%) respondents reported having read the current Brazilian consensus for food allergy (2007) [25] and 80 (19.3%) were aware of some international guideline for food allergy. The European Society for Paediatric Gastroenterology, Hepatology and Nutrition (ESPGHAN) 2012 guidelines [7] was the most known (7.7%).

Among the 16 questions, 10 assessed the adherence to guidelines and awareness of agreement with the recommendations (Table 3). In 8 of these 10 questions, a minority of surveyed pediatricians that answered in accordance with guidelines stated that they knew that their response was in line with the recommendations (20.3–42.3% variation). This finding was statistically significant (p<0.05) in 7 of these 8 questions. The two questions in which the majority consciously adhered to the guidelines were the characterization of risk factors for food allergy (90.8% adherence, with 56.8% conscious adherence (p<0.001)), and the use of extensively hydrolyzed formula as the first option to replace breast milk in cases of CMA (66.5% adherence, with 54.7% conscious adherence (p<0.001). The question that showed the lowest adherence rate was the one that evaluated requiring oral food challenge (OFC) for the diagnosis of CMA, with only 17.8% adherence. This question also showed the lowest conscious adherence, as among those who recommend OFC, only 20.3% were aware of the guideline instruction (p = 0.007). Another question that highlighted poor awareness of guideline adherence was the one about indications to prescribe calcium supplement in CMA (42.9% adherence). In this question, 27.5% of the pediatricians who prescribe calcium appropriately knew that their approach was in compliance with the guidelines.

**Table 3:** Guideline adherence awareness.

Question	Correct answer N (%)	Adherence awareness		*P*-value^a^
		Yes	No or did not know	
Identification of risk factors for food allergy	377 (90.8)	214 (56.8)	163 (43.2)	<0.001
Differentiation between anaphylaxis and FPIES	170 (41.0)	69 (40.6)	101 (59.4)	<0.001
Diagnosis of CMA with late onset gastrointestinal manifestations	241 (58.1)	102 (42.3)	139 (57.7)	0.003
Recognition of FPIAP	207 (49.9)	90 (43.5)	117 (56.6)	<0.001
Recommendation for OFC for diagnosis of CMA	74 (17.8)	15 (20.3)	59 (79.7)	0.007
Timing to OFC (evaluation of tolerance development)	237 (57.1)	81 (34.2)	156 (65.8)	0.076
How is complementary feeding introduced in infants with CMA?	201 (48.4)	74 (36.8)	127 (63.2)	0.010
Appropriate indications for soy formula	141 (34.0)	58 (41.2)	83 (58.8)	0.002
Indication of eHF as the first option to substitute or complement breastmilk in CMA	276 (66.5)	151 (54.7)	125 (45.3)	<0.001
When prescribing calcium supplement in CMA?	178 (42.9)	49 (27.5)	129 (72.5)	0.006

FPIES, Food Protein Induced Enterocolitis Syndrome; CMA, cow´s milk allergy; FPIAP, food protein induced allergic proctocolitis; OFC, oral food challenge; eHF, extensively hydrolyzed formula.

^a^ *p* values were calculated using Chi-square test.

As to the reasons for intentional non-compliance, 14 respondents (3.4%) reported not agreeing with some of the guideline recommendations, 39 (9.4%) followed specific protocols in their workplace where recommendations for the approach of food allergy differ from published guidelines, and 194 (47.7%) reported lack of resources as a reason for not being able to follow guideline recommendations. Some of those surveyed did not give a response to this question and some selected more than one option.

### Adherence analysis

Table 4 shows the factors associated with a level of 80% adherence to guidelines on the care of children with food allergy. The multivariate analysis showed that pediatricians who had read Brazilian consensus (aOR = 2.52; 95%CI = 1.29 to 4.93) and/or were aware of any international guidelines for food allergy (aOR = 2.23; 95%CI = 1.10 to 4.51), or who evaluate more than 10 children per month with suspected CMA (aOR = 2.72; 95%CI = 1.01 to 7.34) were more likely to have an adherence level to the guidelines of ≥80%.

**Table 4:** Univariate and multivariate logistic regression analysis of the variables associated with the adherence rate ≥80% to the guidelines.

VARIABLES	Adherence rate ≥80%		OR (95%CI)	*P*-value	Adjusted OR (95%CI)	*P*-value
	Yes (%)	No (%)				
**Time since conclusion of pediatric residency training (years)**						
0–5	24 (15.5)	131 (84.5)	1.12 (0.63–1.98)	0.70	-	-
6–10	12 (19.0)	51 (81.0)	0.87 (0.42–1.87)	0.71	-	-
>10	33 (17.0)	161 (83.0)	1	-	-	-
**Work setting** **Private medical clinic**						
Yes	38 (17.5)	179 (82.5)	1	-	-	-
No	31 (15.8)	165 (84.2)	1.12 (0.67–1.91)	0.64	-	-
**Public health service**						
Yes	48 (18.2)	216 (81.8)	1.36 (0.77–2.36)	0.28	-	-
No	21 (14.1)	128 (85.9)	1	-	-	-
**Hospital**						
Yes	42 (15.5)	229 (84.5)	1.27 (0.74–2.17)	0.36	-	-
No	27 (19.0)	115 (81.0)	1	-	-	-
**Neonatal service**						
Yes	14 (20.0)	56 (80.0)	1.76 (0.89–3.33)	0.08	1.48 (0.63–3.48)	0.36
No	55 (16.0)	388 (84.0)	1	-	1	
**Previously read Brazilian consensus**						
Yes	39 (27.7)	102 (72.3)	2.51 (1.63–3.85)	<0.001	2.52 (1.29–4.93)	0.007
No	30 (11.0)	242 (89.0)	1	-	-	
**Be aware of any international guideline**						
Yes	25 (31.3)	55 (68.0)	2.36 (1.55–3.62)	<0.001	2.23 (1.10–4.51)	0.02
No	44 (13.2)	289 (86.8)	1	-	-	
**Evaluation of children with suspected CMA**						
Yes	56 (18.6)	245 (81.4)	1.73 (0.92–3.43)	0.09	1.35 (0.97–3.38)	0.35
No	13 (11.6)	99 (88.4)	1	-	1	-
**Number of children with suspected CMA evaluated per month**						
0–5 children	31 (15.0)	175 (85.0)	1	-	1	-
6–10 children	12 (23.5)	39 (76.5)	1.73 (0.79–3.64)	0.14	1.83 (0.59–5.63)	0.16
>10 children	9 (39.1)	14 (60.9)	3.60 (1.38–9.08)	0.003	2.72 (1.01–7.34)	0.04

OR, odds ratio; CI, confidence interval; CMA, cow´s milk allergy

## Discussion

Although international CPG for food allergy in children have been published, providing recommendations for the diagnosis and management of this disorder based on scientific evidence [7–10,12–16], Brazilian pediatricians self-reported adherence to their recommendations is low. Our findings highlight that better adherence to guidelines is associated with more frequent evaluation of children with suspected cow’s milk allergy, reading of Brazilian food allergy consensus and awareness of any international food allergy guidelines.

Recent studies have shown that adherence to guidelines by practitioners improves health indicators [27]. However, adherence to guidelines is usually poor, despite all efforts made in their implementation [28]. There are several international food allergy guidelines [7–10,12–16], as well as Brazilian consensuses [25,29,30], but there are as yet few studies that evaluate the adherence of pediatricians to these recommendations [23,31,32]. As our questionnaire evaluated adherence to international guidelines, it may be used elsewhere without restriction.

The rates of adherence of Brazilian pediatricians to food allergy management guidelines were low. No pediatrician surveyed had a 100% adherence rate, and only 16.7% showed a rate equal to or greater than 80%. However, these results far outweigh the one evaluating adherence to the 2009 NASPGHAN-ESPGHAN guideline for gastroesophageal reflux, where only 0.5% of the respondents had an adequate compliance rate [33]. This may be explained by the fact that for food allergy there are several different guidelines, making knowledge on the subject easier to propagate, but when we evaluated the surveyed responses, we found that only 34% of participants had read the Brazilian consensus for food allergy and only 19.3% were aware of any international guidelines. So, despite the existence of a number of guidelines, lack of awareness is a significant barrier to guideline adherence. Gaps in the knowledge of Brazilian pediatricians about the treatment of CMA, the major food allergy in infants in our country, have been demonstrated previously [24], but opportunities are present in Brazil for pediatricians to gain knowledge regarding the approach to food allergy. Ensuring pediatricians have a good understanding of food allergy management is crucial for children with suspected CMA as they are mostly managed by general pediatricians in Brazil, without OFC to confirm diagnosis.

In the multivariate analysis, the variables ‘having read the Brazilian consensus’, ‘being aware of any international guideline’ and ‘evaluating more than 10 children with suspected CMA per month’ were the statistically significant variables for an adherence rate of ≥ 80%, but there was no association between evaluating more children and having being aware of some guideline. The relationship between knowledge of recommendations and practice in accordance with recommendations is described in the literature assessing the knowledge and practice of pediatricians in New York City, USA, against guidelines for two clinical conditions common in children—bronchiolitis and community-acquired pneumonia (CAP). They found that lack of knowledge was associated with the prescription of unnecessary treatments [34]. Guideline adherence is a multifactorial outcome. Although being aware of the recommendations improve adherence, other variables not investigated such as continuous education, academic degree and others must act as confounding factors.

Some studies that focused on prevention rather than treatment of food allergy have assessed adherence to recommendations and also reported low adherence. A Brazilian study reported that 41.9% of pediatricians and nutritionists recommended delayed introduction of allergenic foods in allergic infants [23]. In 2017, Vandenplas *et al*. [32] reported a similar finding when they evaluated the adherence of 1,481 physicians (66.1% pediatricians and 7,1% pediatric gastroenterologists) from Middle Eastern and North African countries regarding primary prevention of food allergy, and 60% recommended delayed introduction of potentially allergenic foods. In our study, 51.6% of those surveyed did not adhere to the guidelines regarding the introduction of complementary feeding to children already diagnosed with CMA, and most of them did not know if this practice complied with the guidelines.

Guideline adherence has been discussed by many authors worldwide in the past decades. Cabana and colleagues (1999) [35] identified a wide spectrum of barriers to guideline adherence such as lack of awareness, lack of familiarity, lack of agreement, lack of self-efficacy, lack of outcome expectancy, inertia of previous practice and external barriers that impact guideline implementation. Intentional non-compliance may be motivated by valid reasons, mainly related to contraindications and patient preferences, they must be considered when developing a guideline [36], but lack of awareness seems to be an important barrier in our study. A large study conducted in the USA showed that only 55% of patients are cared for according to the recommendations described in guidelines [37]. The barriers to adherence may be related to health care professionals but also to patients, to the organizational context and the social and cultural context of the health care system [38].

In the present study, most respondents had not read the Brazilian consensus or were not aware of any international guidelines and a minority knew that their response was in agreement with the recommendations in 8 of 10 questions that assessed this aspect. The reasons for intentional lack of adherence were questioned, and the most frequent answer was the lack of resources to implement the recommendation. However, there was no statistically significant difference among those surveyed from the five regions of the country. Considering that Brazil is a large country with profound economic inequalities within different regions, we cannot clearly explain this finding. However, it is known that many of the interregional inequalities may also be present within the same region in the country [39], which may help to explain the similar findings in responses from pediatricians practicing in different parts of the country.

An essential point to consider in this analysis is the quality of the available guidelines, which vary significantly and may compromise adherence. In 2016, Ruszczynski et al. [11] evaluated 15 CMA guidelines using the AGREE II instrument. Of these, only 2 [8,12] reached the highest score (overall quality 100%), and 8 were considered high quality (overall quality >60%). Among the domains evaluated for each guideline, applicability had the lowest mean score. The quality of the evidence might have an impact on adherence as described by O’Sullivan and colleagues (2018) [40]. Considering that Brazilian recommendations are published based on a consensus, we only investigated the adherence to high quality guidelines according to Ruszczynski et al. (2016) [11] and that were in agreement with the national consensus and international guidelines to exclude quality of recommendation as a bias.

Editorial independence is one of the AGREE II domains and must be ensured during guideline development [41]. Experts usually develop guidelines and have a potential conflict of interest because of professional involvement with the pharmaceutical industry. In the study by Ruszczynski et al. (2016) [11], 6 in 15 food allergy guidelines did not reach a satisfactory score in this domain.

Adequate diagnosis and management of food allergy in children is essential to avoid unnecessary treatments and to ensure adequate growth and development, quality of life and rational use of financial resources [1,42,43]. Therefore, it is necessary to develop strategies such as educational activities, increased multidisciplinary work, performance evaluation and social influence to ensure guideline implementation [44].

Our study has some limitations. We investigated the self-reported adherence using a questionnaire without an *in situ* confirmation; however, this type of survey has never been done before in Brazil, and the method of data collection we used allowed us to collect a significant number of responses from pediatricians from different regions of Brazil. It is possible that basing the study on a non-probabilistic sample of Brazilian pediatricians who were attending a congress could have led to bias as the pediatricians surveyed might be more interested in up-to-date information. However, even among these pediatricians we found low adherence to guideline recommendations. Thus, we may expect that adherence in the broader population and in real scenarios might be even lower and represent an even more serious problem than our data presented here suggest. We invited pediatricians attending a congress and did not register some refusal to participate as well as we did not investigate surveyed pediatric specialty, they are also limitations once we may have non-response and response bias. However, we surveyed during a general pediatrician congress and if some surveyed have a pediatric specialty related to food allergy it could improve results, so real life data among general pediatricians must be even worse than we observed.

The lack of resources to implement the recommendation was the most reported barrier to adherence among respondents, probably because difficulty in performing OFC and/or availability of high cost hypoallergenic formulas (extensive hydrolyzed formula and amino acid based formula) in a country where a large portion of population do not have financial resources to support basic necessities, but there were no statistically significant differences when comparing the economically different regions. It seems that the lack of awareness plays the most important role, since low adherence occurs even in high-income countries and, here, most of those surveyed were not aware of the recommendations. Another limitation was not investigating the perception of those surveyed in respect of guideline quality and its relation with adherence. Considering that most pediatricians demonstrated a lack of awareness of the guidelines, we infer that this limitation had a low impact on our results.

Although international CPG for food allergy in children have been published, providing recommendations for the diagnosis and management of this disorder based on scientific evidence [7–10,12–16], Brazilian pediatricians self-reported adherence to their recommendations is low. Our findings highlight that better adherence to guidelines is associated with more frequent evaluation of children with suspected cow’s milk allergy, reading of Brazilian food allergy consensus and awareness of any international food allergy guidelines.

We observed a low self-reported adherence to food allergy guidelines among Brazilian pediatricians and this is the first countrywide guideline adherence assessment for this pathology. The lack of awareness of the recommendations is related to the low adherence, as well as to the evaluation of a smaller number of children with suspected CMA, the major cause of food allergy in Brazilian infants. Good quality guidelines, as well as studies to evaluate efficacy of strategies for adherence, are required to improve their implementation. As this questionnaire is based on international guidelines, it may be useful in evaluating adherence in other countries.

## Supporting information

S1 File Questionnaire. Food Allergy Management in Children and Adherence to Guidelines.
(PDF)

S2 File Study data table. (XLSX)
