# Supplementary material for: Brazilian pediatricians’ adherence to food allergy guidelines—A cross-sectional study
Source: PLoS One. 2020 Feb 24;15(2):e0229356. doi: 10.1371/journal.pone.0229356 (PMC7039437; doi:10.1371/journal.pone.0229356)
Supplement: S1 File — Food Allergy Management in Children and Adherence to Guidelines. (PDF) [file pone.0229356.s001.pdf]

S1 File. Questionnaire

SURVEY
Food Allergy Management in Children
and Adherence to Guidelines

Dear colleague, this questionnaire presents questions about your knowledge and practice in relation to food allergy. In respect of the questions about practice, the expectation is that your response is in line with what you usually do, even if it is not what is stated in the guidelines and publications. It is very important to know what exactly is done in practice. We remind you that the results will be analyzed anonymously.

1. In addition to allergic rhinitis, asthma and food allergy, which of the following conditions, when present in parents or siblings, should be considered to characterize high risk of food allergy?

Objective: Identification of risk factors for food allergy

a. drug allergy
b. chronic urticaria
c. atopic dermatitis
d. contact dermatitis

Does your answer above comply with the guidelines?
( ) Yes ( ) No ( ) I don't know

2. Cow's milk allergy is an adverse reaction to:

a. lactose - immune reaction
b. lactose - pharmacological reaction
c. cow's milk proteins - immunological reaction
d. cow's milk proteins - pharmacological reaction

3. Among the situations described below, you suspect anaphylaxis to cow's milk in, EXCEPT:

Objective: Differentiation between anaphylaxis and FPIES

a. four-month-old infant that presents urticaria and bronchospasm 30 minutes after the first ingestion of cow's milk-based formula
b. five-year-old child with an established diagnosis of cow's milk allergy who presents only hypoactivity and hypotension 10 minutes after milk ingestion
c. one-year-old child presenting urticaria and 3 episodes of vomiting 1 hour after the ingestion of lactose-free milk
d. six-month-old child with vomiting (between 1-4 hours), and diarrhea, dehydration and lethargy (5-10 hours) after ingestion of natural cow's milk

Does your answer above comply with the guidelines?
( ) Yes ( ) No ( ) I don’t know

4. Regarding the diagnosis of CMA (cow’s milk allergy) with delayed reaction and gastrointestinal manifestation it is CORRECT to state that:

Objective: Diagnosis of CMA with late onset gastrointestinal manifestations

a. a favorable clinical response to a milk and dairy elimination diet is sufficient, dispensing with the need for oral food challenge
b. clinical history plays a key role in requiring laboratory tests and/or oral food challenge
c. cow’s milk proteins specific IgE (*in vivo* and/or *in vitro*) must be performed
d. a favorable clinical response to a milk and dairy elimination diet associated with positive stools tests (e.g. fecal occult blood test, leukocytes) is sufficient

Does your answer above comply with the guidelines?
( ) Yes ( ) No ( ) I don’t know

5. An exclusively breastfed three-month-old infant with good overall health status and adequate weight gain, but with mucus and bloody diarrhea that disappear after maternal milk and dairy elimination diet. In this case, there is suspicion of CMA manifested as:

Objective: Recognition of FPIAP (food protein induced allergic proctocolitis)

a. food protein induced enterocolitis syndrome (FPIES)
b. food protein induced enteropathy (FPE)
c. food protein induced allergic proctocolitis (FPIAP)
d. none of the above

Does your answer above comply with the guidelines?
( ) Yes ( ) No ( ) I don’t know

6. When do you order the oral food challenge (OFC):

In most cases, to confirm or exclude the diagnosis
( ) Yes ( ) No ( ) No answer

I usually wait until the child consumes cow’s milk protein in daily life.

Objective: Recommendation of OFC for diagnosis of CMA

( ) Yes ( ) No ( ) No answer

I usually don´t order OFC either for diagnostic purposes or for tolerance evaluation (food allergy cure)

( ) Yes ( ) No ( ) No answer

In most cases, to evaluate tolerance (food allergy cure) after an elimination diet.

Objective: Timing to OFC (evaluation of tolerance development)

( ) after 6-12 months depending on the clinical condition
( ) at 1 year old
( ) no response

Does your answer above comply with the guidelines?
( ) Yes ( ) No ( ) I don't know

7. If you perform the oral food challenge, in which setting do you do it?

a. home
b. as an outpatient or inpatient
c. always as an inpatient
d. home, as an inpatient or outpatient depending on the pattern of clinical reactions and the immunological mechanism involved

8. For infants in CMA treatment, how do you manage complementary feeding introduction?

Objective: How is complementary feeding introduced in infants with CMA?

a. as recommended for healthy children
b. by excluding all potentially allergenic foods (egg, seafood, etc.) up to 1 year old
c. after 6 months of age, but I do not introduce meat because of the risk of cross-reaction
d. after 6 months of age, but I maintain the elimination of potentially allergenic foods until oral tolerance to cow's milk is achieved (milk allergy cure)

Does your answer above comply with the guidelines?
( ) Yes ( ) No ( ) I don't know

9. Do you use isolated soy protein-based formula for the treatment of cow's milk protein allergy?

Objective: Appropriate indication for soy formula

a. as first choice for IgE or non-IgE mediated allergy, regardless of age
b. for infants over 6 months of age with IgE mediated allergy
c. for infants over 6 months of age with non-IgE mediated allergy
d. none of the above

Does your answer above comply with the guidelines?
( ) Yes ( ) No ( ) I don't know

10. For you, what is the reason for indicating isolated soy protein-based formula for a child with CMA?

a. price
b. efficiency
c. palatability
d. I do not use it

11. What is your first option to substitute or complement breastmilk in CMA?

Objective: Indication of extensively hydrolyzed formula as the first option to substitute or complement breastmilk in CMA

a. partially hydrolyzed whey infant formula (pHF)
b. lactose-free milk protein-based infant formula
c. extensively hydrolyzed infant formula depending on clinical condition severity
d. free amino acid-based formula, regardless severity

Does your answer above comply with the guidelines?
( ) Yes ( ) No ( ) I don't know

12. Which of the following are limiting factors on the choice of substitute to special formulas for treatment of CMA over 2 years old?

a. homology between the proteins of goat's milk and cow's milk
b. high protein content in rice milk
c. high probability of concomitant reaction of soy milk and cow's milk
d. all of the above

13. When managing milk and dairy elimination diet, you should prescribe calcium supplement to all those below except one, which one?

Objective: When prescribing calcium supplement in CMA?

a. breastfeeding mothers who are on milk and dairy elimination diet
c. one-year-old child, depending on the formula intake
c. any age children with CMA diagnosis, regardless diet composition
d. two-years-old child on diet without formula

Does your answer above comply with the guidelines?
( ) Yes ( ) No ( ) I don't know

14. Have you read the Brazilian Consensus on Food Allergy (2007)?
( ) Yes ( )No

15. Do you know any international food allergy guideline?
( ) Yes ( ) No. Which one?
____

16. In case of intentional disagreement, what is the reason? (You can check more than one option)

a. lack of resources to put the recommendation into practice
b. I do not agree with the recommendation
c. the recommendation of my work setting differs from the recommendation of the consensus or guideline

d. none of the above

Explain:____
____
____
